# Copy Number Variation of Fc Gamma Receptor Genes in HIV-Infected and HIV-Tuberculosis Co-Infected Individuals in Sub-Saharan Africa

**DOI:** 10.1371/journal.pone.0078165

**Published:** 2013-11-08

**Authors:** Lee R. Machado, Jennifer Bowdrey, Eliford Ngaimisi, Abiy Habtewold, Omary Minzi, Eyasu Makonnen, Getnet Yimer, Wondwossen Amogne, Sabina Mugusi, Mohammed Janabi, Getachew Aderaye, Ferdinand Mugusi, Maria Viskaduraki, Eleni Aklillu, Edward J. Hollox

**Affiliations:** 1 Department of Genetics, University of Leicester, Leicester, United Kingdom; 2 Division of Clinical Pharmacology, Department of Laboratory Medicine, Karolinska Institutet, Stockholm, Sweden; 3 Unit of Pharmacology, School of Pharmacy, Muhimbili University of Health and Allied Sciences, Dar es Salaam, Tanzania; 4 Department of Pharmacology, Addis Ababa University, Addis Ababa, Ethiopia; 5 Internal Medicine, Addis Ababa University, Addis Ababa, Ethiopia; 6 Institution of Medicine, Unit of Infectious Diseases, Karolinska Institutet, Karolinska University Hospital, Huddinge, Sweden; 7 Department of Internal Medicine, Muhimbili National Hospital, Dar es Salaam, Tanzania; 8 Department of Internal Medicine, Muhimbili University of Health and Allied Sciences, Dar es Salaam, Tanzania; 9 College of Medicine, Biological Sciences and Psychology, University of Leicester, Leicester, United Kingdom; University of Texas Health Science Center San Antonio Texas, United States of America

## Abstract

AIDS, caused by the retrovirus HIV, remains the largest cause of morbidity in sub-Saharan Africa yet almost all genetic studies have focused on cohorts from Western countries. HIV shows high co-morbidity with tuberculosis (TB), as HIV stimulates the reactivation of latent tuberculosis (TB). Recent clinical trials suggest that an effective anti-HIV response correlates with non-neutralising antibodies. Given that Fcγ receptors are critical in mediating the non-neutralising effects of antibodies, analysis of the extensive variation at Fcγ receptor genes is important. Single nucleotide variation and copy number variation (CNV) of Fcγ receptor genes affects the expression profile, activatory/inhibitory balance, and IgG affinity of the Fcγ receptor repertoire of each individual. In this study we investigated whether CNV of *FCGR2C*, *FCGR3A* and *FCGR3B* as well as the HNA1 allotype of *FCGR3B* is associated with HIV load, response to highly-active antiretroviral therapy (HAART) and co-infection with TB. We confirmed an effect of TB-co-infection status on HIV load and response to HAART, but no conclusive effect of the genetic variants we tested. We observed a small effect, in Ethiopians, of *FCGR3B* copy number, where deletion was more frequent in HIV-TB co-infected patients than those infected with HIV alone.

## Introduction

AIDS, caused by the T-lymphotropic retrovirus HIV, remains the largest cause of morbidity in sub-Saharan Africa [1]. African countries currently have the highest disease burden of HIV, with 9.2% prevalence in Addis Ababa in Ethiopia and over 10% in Dar-es-Salaam in Tanzania, yet almost all genetic studies have focused on cohorts from Western countries [2]. In Africa, HIV shows high co-morbidity with tuberculosis (TB), as HIV stimulates the reactivation of latent TB, and we and others have shown that TB co-infection is associated with a higher viral load (VL) prior to treatment and a poorer response to treatment [3-5] . This presents challenges to the standard treatment regimens of both HIV and TB [6,7]. 

The most effective treatment for HIV and TB would be an effective vaccine; several are currently in clinical trials for HIV (e.g., STEP trial, RV144), and for TB, the vaccine Bacillus Calmette-Guérin (BCG) remains ineffective against pulmonary TB in adults. An effective vaccine is likely to stimulate the production of potent broadly neutralising antibodies that are able to neutralise the pathogen. However, in the recent RV144 trial, when looking for correlates of protection from HIV-1 infection, it was found that neutralising antibodies and cytotoxic T lymphocyte (CTL) responses were absent in protected patients [8]. In contrast, HIV -1 infection inversely correlated with gp120 V1V2-specific and antibody-dependent cell cytotoxicity (ADCC)- and antibody-dependent cell-mediated viral inhibition (ADCVI)-mediating non-neutralising antibodies. Given that Fcγ receptors are critical in mediating the non-neutralising effects of antibodies, this suggests an important role for Fcγ receptors in recruiting innate immune cells to sites of HIV infection. The interaction between the Fc region of IgG and Fcγ receptors is critical for mediating the biological effects of the humoral immune response, such as ADCC and ADCVI. The ratio of activatory/inhibitory signals generated by engagement of different Fcγ receptors by IgG determines the threshold for induction of IgG-mediated responses. In addition, it has been shown that Fcγ receptor function is critical in mounting an effective response to HIV infection in experimental animals [9].

Fcγ receptor genetic variation has been associated with infectious and inflammatory disease in both genomewide [10] and candidate gene studies [11,12], and it is known that at least some of this variation affects function, both in terms of subcellular localisation, cell type expression and IgG subtype affinity binding [13,14]. Two studies have suggested that genetic variation of host Fcγ receptors may affect various aspects of HIV infection and progression. The *FCGR2A* gene encodes an activating receptor expressed on macrophages and neutrophils, and a coding polymorphism (rs1801274 c.497AG [p.His131Arg]) has been associated with susceptibility to perinatal HIV infection [15] and variation in HIV progression to AIDS [16], with His131 homozygotes showing increased perinatal transmission and more rapid progression to AIDS. It is known that the two different alleles differ markedly in their affinity for IgG2 [13,17], and it was shown not only that anti-gp120 IgG2 complexes were present in individuals chronically infected with HIV, but that HIV-1 immune complexes were internalised more efficiently by monocytes from donors who were homozygous for the His131 allele. 

Initial studies have focused on two alleles of one gene, but the genetic variation of the FCGR region is extensive and complex. In particular, the *FCGR3A* and *FCGR3B* genes are 97% identical and the product of an 80kb duplication that occurred after the divergence between macaque and human-chimpanzee lineages (~25 million years ago) [18]. They encode two different Fcγ receptors, with *FCGR3A* being expressed on natural killer (NK) cells, monocytes, dendritic cells, and macrophages and *FCGR3B* being expressed on neutrophils, mast cells, and eosinophils. Both genes exhibit copy number variation, deletion of the *FCGR3B* gene is associated with both SLE and RA, and is likely to cause ectopic expression of the *FCGR2B* inhibitory receptor on NK cells [19,20]. The *FCGR2C* gene is an activating receptor, formed as a fusion gene of *FCGR2A* and *FCGR2B* during the duplication of the ancestral *FCGR3* gene and is expressed on NK cells. *FCGR2C* shows copy number variation related to the copy number variation of *FCGR3A* and *FCGR3B*, such that deletion or duplication of *FCGR3A* or *FCGR3B* results in concomitant deletion or duplication of *FCGR2C*. There is also the additional complication of a variant (rs10917661; c.169CT) which converts a glutamine to a stop codon, rendering *FCGR2C* non-functional [21]. *FCGR3B* also carries two major alleles that produce isoforms differing by 4 amino acids called human neutrophil antigen 1a and 1b (HNA1a and HNA1b ; rs76714703)[22] . These alleles affect binding to IgG1 and IgG3 and phagocytosis of opsonized particles.

In this study we investigated whether copy number variation of *FCGR2C*, *FCGR3A* and *FCGR3B* as well as HNA1 allelic variation of *FCGR3B* is associated with HIV load, response to HAART and co-infection with TB in two African populations.

## Methods

### Samples and ethics statement

Patient sample and clinical data collection was as previously described [5,23]. DNA extraction was performed using QIAamp DNA Maxi kit in a single laboratory. The study protocol was approved by the Institutional Review Board at the Faculty of Medicine, Addis Ababa University and Ethiopian Science and Technology Ministry; the regional ethical review board in Stockholm at the Karolinska Institutet and the ethical review committee of Muhimbili University of Health and Allied Sciences. Written informed consent was obtained from each subject before the start of this study. All samples had previously been shown to be homozygous for the *CCR5* 32bp insertion allele [5], where the deletion allele is known to be protective against HIV progression. The breakdown of samples analysed is shown in Table 1.

**Table 1:** Summary of samples analysed.

		Ethiopian	Tanzanian
Samples analysed	n	720	347
Samples analysed with detailed clinical data	n	618	347
*FCGR3* copy number genotypes and detailed clinical data	n	607	344
CD4<200 with baseline VL data and genotypes	n	517	167
	males	201	72
	females	316	95
	CD4 (mean +/- sd)	92.30 +/-52.978	93.33 +/- 61.274
	VL x 10^5^ (mean +/- sd)	3.32 +/- 6.78	4.87 +/- 10.66
CD4<200 with at least one CD4 follow-up datapoint for response analysis	n	373	137
	males	128	59
	females	256	78
	CD4 (mean +/- sd)	97.89 +/-52.835	97.05 +/- 60.252
	VL x105 (mean +/- sd)	2.98 +/-5.43	4.58 +/- 8.44

### Copy number analysis

**Figure 1:**
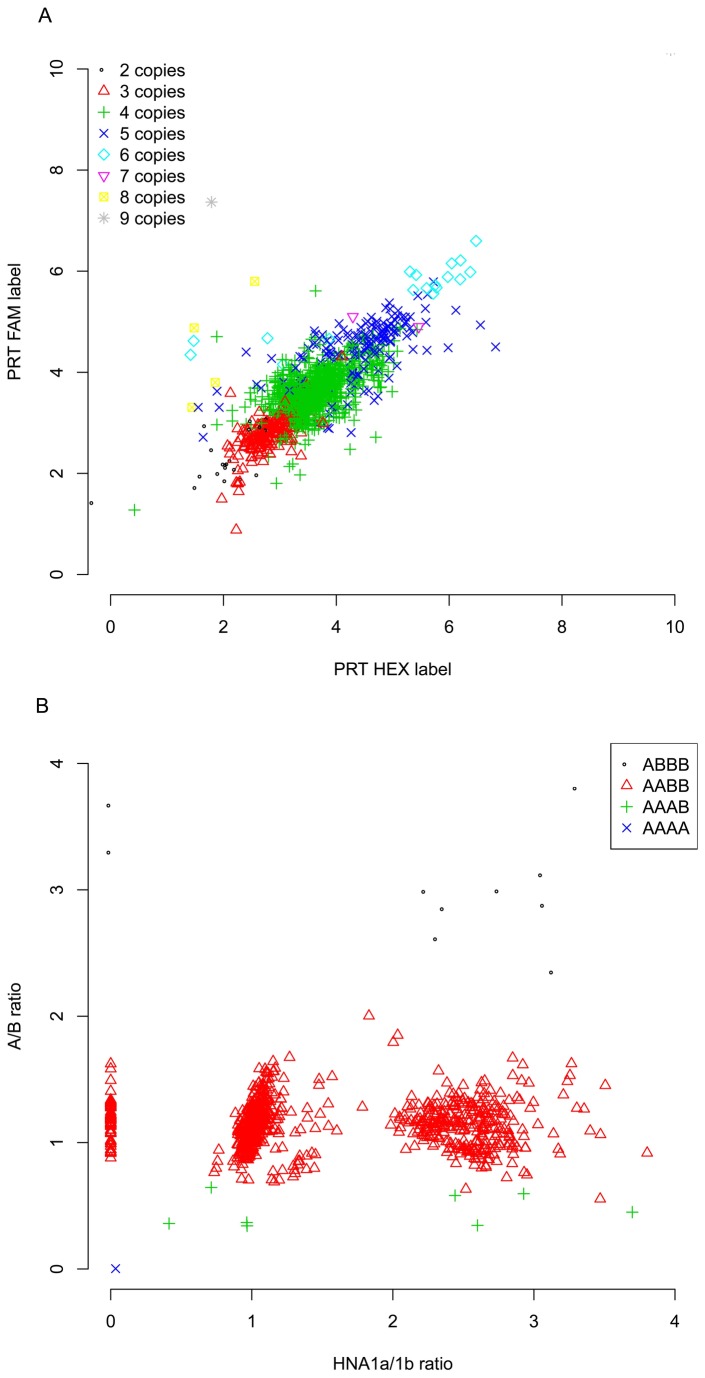
Analysis of raw copy number quantification data. *a*) Correlation between individual PRT copy number estimates. Copy number typing uses duplicate PRTs combined with restriction enzyme digest ratios to infer copy number. Individual raw PRT results from the FAM-labelled experiment (y-axis) and the HEX-labelled experiment are plotted for all samples, colour-coded according to the estimated integer copy number of each sample. *b*) *Analysis* *of* *restriction* *enzyme* *digest* *ratios* . Raw ratios for the A/B assay and the HNA1a/1b assay are plotted for all samples with a total *FCGR3* copy number estimate of 4. Each point is coloured according to the *FCGR3A*:*FCGR3B* ratio estimate.

Copy number analysis was performed as described previously [18]. Briefly, duplicate calls from a paralogue ratio test (PRT, [24]) are combined with three independent assays measuring restriction enzyme digestion ratios using a maximum-likelihood framework, which calls the most-likely integer copy number given the results from the five assays. Raw data from the duplicate PRTs show very strong concordance with limited clustering and a small number of outliers (Figure 1a), showing that duplicate PRT by itself is not sufficient to reliably call copy number in all samples, and the extra information provided by restriction enzyme variant ratios (REDVR) is needed. The raw results for two REDVR assays are shown in Figure 1b. The two assays distinguish *FCGR3A* from *FCGR3B* using the arginine to stop change (C733T, y axis) and distinguish HNA1a and HNA1b on *FCGR3B* using the C147T nonsynonymous change [22,25,26]. Clear clustering of samples is observed, and the samples are classified according to the number of *FCGR3A* and *FCGR3B* copies observed. Note that, for clarity, only samples with four copies of *FCGR3* (*FCGR3A* and *FCGR3B*) are shown.

**Figure 2:**
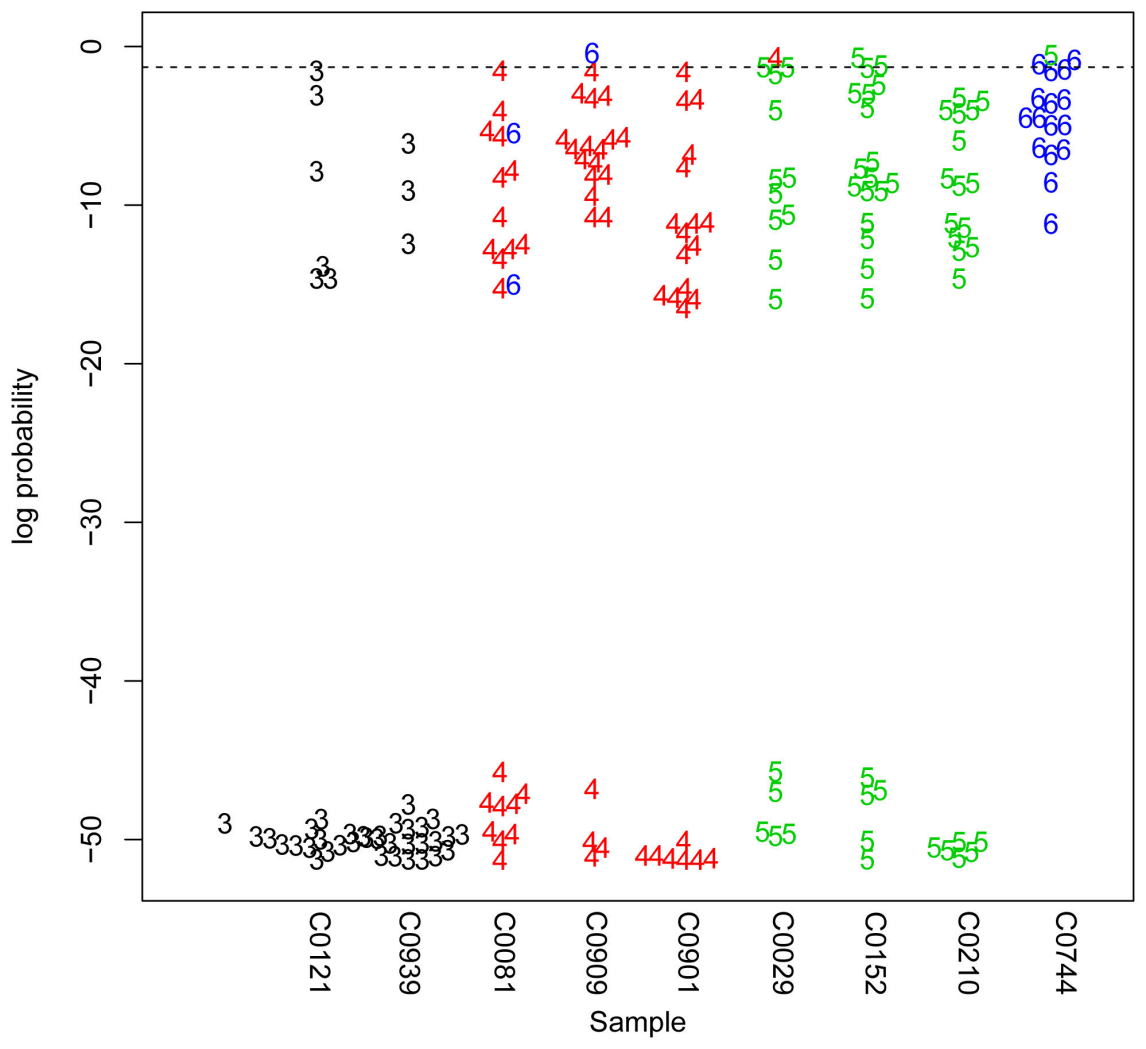
Positive control total *FCGR3* copy number estimates from repeat tests. The nine positive control DNA samples shown were repeated in every experiment and total *FCGR3* copy number calculated. Each point represents an individual copy number measurement (as indicated by the colour and number), plotted on the y-axis representing the quality score as a log probability of the copy number being an alternative to the copy number shown. The dotted line shows the quality score threshold of 0.05, with samples below this threshold being accepted. The lower the point on the y-axis, the more confident we are of the copy number. 190 tests are shown, with 183 being below the quality threshold. Of these 183, 2 show the incorrect copy number, both in C0081 calling a 6 instead of a 4.

Integer copy numbers were inferred from all assays combined using the maximum-likelihood approach described previously [25], which generates an associated quality score for each copy number call. As previously, copy number estimates with a quality score of p<0.05 (equivalent to an odds ratio of 20:1 of this copy number being correct against any other copy number being correct). Positive control samples from the Human Random Control collection (Health Protection Agency, Salisbury, UK) were run with every experiment and provided a measure of the reproducibility of the assay (Figure 2). Of 190 repeat tests, 183 (96%) passed the quality score threshold. Of these 183, 2 (1%) gave the incorrect copy number score, suggesting an error rate for total copy number of 1-2%. Individual gene copy numbers were inferred using the total copy number score and the observed ratio value of the particular assay. For example, from a 1:1 ratio of the *FCGR3A*:*FCGR3B* REDVR with a total copy number of 4, we would infer a copy number of 2 for *FCGR3A* and 2 for *FCGR3B*.

### Statistical analysis

To analyse the effect of genotype on HIV load at initiation of HAART, we initially constructed a generalised linear model using SPSS 20.0 (IBM) and a gamma-log link, using Type III sum of squares and Wald estimation. Initial VL was used as the dependent variable, with population and disease status as fixed predictor factors, CD4 count and genotype as scalar predictor variables. 

To examine the effect of genotype on CD4+ count following initiation of HAART, we constructed a generalised linear mixed model, using STATA, where the dependent variable (CD4+ count) was modelled as a Gaussian distribution. In this model, we assigned population and disease status as fixed factors, initial CD4 count and time since HAART initiation as scalar covariates and integer copy number as an ordinal covariate. The model was calculated using Type III sum of squares, with a variance correction to allow for multiple CD4+ timepoint readings from a single patient.

Two-tailed t-tests were performed in Microsoft Excel, assuming unequal variances of the two samples.

## Results and Discussion

### Distribution of copy number alleles

Previous studies have shown that the deletion and duplication alleles for both *FCGR3A* and *FCGR2B* are relatively rare in the population [18,25]. Our data support this: in both Tanzanian and Ethiopian populations the frequency of heterozygotes for deletions and duplications is approximately 5%, and the frequency of homozygous deletions or duplications is less than 1% (table 2). 

**Table 2:** *FCGR3A* copy number frequencies.

copy number	Ethiopian HIV Count (frequency)	Ethiopian HIV+TB Count (frequency)	Tanzanian HIV Count (frequency)	Tanzanian HIV+TB Count (frequency)
0	0 (0)	1 (<0.01)	0 (0)	0 (0)
1	13 (0.05)	24 (0.05)	4 (0.02)	20 (0.14)
2	249 (0.90)	390 (0.88)	186 (0.92)	121 (0.83)
3	15 (0.05)	23 (0.05)	12 (0.06)	3 (0.02)
4	1 (<0.01)	4 (<0.01)	0 (0)	1 (<0.01)
5	0 (0)	0 (0)	0 (0)	0 (0)
total	278	442	202	145
mean	2.01	2.01	2.04	1.91

Both the *FCGR3B* deletion allele and the *FCGR2C* active allele have a functional effect. For carriers of the *FCGR3B* deletion allele, not only is there a gene dosage effect resulting in a lower expression of *FCGR3B* on the surface of neutrophils [27], but a change in the expression pattern of the adjacent *FCGR2B* gene, which is not copy number variable itself. The *FCGR2B* gene encodes the only known inhibitory Fc gamma receptor, and deletion of *FCGR3B* results in ectopic expression of *FCGR2B* on NK cells, possibly as a result of a NK-specific regulatory element being brought into closer physical proximity to the *FCGR2B* gene [19,20]. Again, as observed previously, the frequency of deletions and duplications of the *FCGR3B* gene is higher than for the *FCGR3A* gene (table 3),

**Table 3:** *FCGR3B* copy number frequencies.

copy number	Ethiopian HIV Count (frequency)	Ethiopian HIV+TB Count (frequency)	Tanzanian HIV Count (frequency)	Tanzanian HIV+TB Count (frequency)
0	1 (<0.01)	9 (0.02)	0 (0)	0 (0)
1	38 (0.14)	69 (0.16)	20 (0.10)	28 (0.19)
2	177 (0.64)	306 (0.69)	166 (0.82)	109 (0.75)
3	57 (0.21)	54 (0.12)	16 (0.08)	6 (0.04)
4	4 (0.01)	3 (<0.01)	0 (0)	1 (<0.01)
5	0 (0)	1 (<0.01)	0 (0)	1 (<0.01)
6	1 (<0.01)	0 (0)	0 (0)	0 (0)
7	0 (0)	0 (0)	0 (0)	0 (0)
total	278	442	202	145
mean	2.10	1.95	1.98	1.88

The *FCGR2C* gene encodes an activating receptor that is normally expressed on NK cells [21]. Not only is *FCGR2C* copy number variable but there is a common variant (rs10917661; p. Q57*; c.169CT) which results in a pseudogene, so that, in Europeans, most people do not express *FCGR2C*. In this study, we combine measurement of *FCGR2C* copy number together with detection of the allelic status of *FCGR2C* to determine, for each individual, the number of copies of *FCGR2C* that are predicted to encode a full-length functional gene. We show there is an appreciable frequency of the active *FCGR2C* allele in both African populations tested (table 4), although we note that this may be an overestimate, as we did not determine the “non-classical” *FCGR2C* null allele, likely to be caused by a single nucleotide variant in the donor splice site of intron 7, causing skipping of exon 7 and subsequent frameshift causing early termination of the polypeptide chain [19].

**Table 4:** *FCGR3C* (active) copy number frequencies.

copy number	Ethiopian HIV Count (frequency)	Ethiopian HIV+TB Count (frequency)	Tanzanian HIV Count (frequency)	Tanzanian HIV+TB Count (frequency)
0	211 (0.76)	375 (0.86)	162 (0.81)	128 (0.88)
1	59 (0.21)	54 (0.12)	36 (0.18)	14 (0.10)
2	6 (0.02)	7 (0.02)	1 (<0.01)	3 (0.02)
total	276	436	199	145
rs10917661 Q57 allele frequency	0.13	0.08	0.10	0.07
rs10917661 Q57 allele count	71	68	38	20
rs10917661 *57 allele count	481	804	360	270

We initially compared the mean copy number between the HIV-only and HIV-TB co-infected cohorts in both Tanzanians and Ethiopians. While there was no significant difference between *FCGR3A* copy number (table 2), we did find a lower mean copy number for *FCGR3B* in the HIV-TB co-infected cohort in both populations, which was significant in the Ethiopian cohort (table 3, two-tailed t-test p=0.002). Although not significant in the smaller Tanzanian cohort (two-tailed t-test p=0.078), a striking frequency difference in *FCGR3B* 1 copy individuals is observed in the Tanzanian HIV only (10%) and HIV-TB (19%) cohorts. We also observe a lower mean active copy number for *FCGR2C* in the HIV-TB co-infected cohort in both populations (table 4), which was significant again only in the Ethiopian cohort (two-tailed t-test p=0.004), which is expected given the close relationship between *FCGR3B* and *FCGR2C* copy number. Taken together, this might suggest greater expression of inhibitory over activatory FCGR2 receptors on NK cells is associated with co-infection of TB with HIV. Indeed, the role of Fc receptors and NK cells in TB is unclear and under-explored, with most work focused on the cellular T-cell-mediated response. In mice NK cells respond to *Mycobacterium tuberculosis* infection yet are not critical in protection [28], but deletion of the inhibitory Fc receptor *FCGR2B* expressed on B cells resulted in more effective mycobacterial containment [29]. Although the Ethiopian cohort is larger than the Tanzanian cohort, and therefore has more power to detect a significant effect, our data should be interpreted cautiously, as our observation was only significant in the Ethiopian cohort, and the HIV-TB coinfected and HIV-only cohorts were different arms of the study and quantitative differences may be due to subtle batch effects. The observed effect size is on the edge of the size of effect that the study is powered to predict with the Ethiopian cohort having 80% power to predict a difference in means of 0.14, and the Tanzanian cohort having 80% power to predict a difference in means of 0.15. At the moment, we suggest that this is an intriguing result that awaits further study.

### Association of Fcγ receptor copy number and allelic variation with baseline viral load and progression post HAART administration

 We then analysed the effect of the allelic variation of copy number of the three genes, and HNA1 allelic status, on HIV load just prior to initiation of HAART in patients whose CD4 count was less than 200. As previously published [5], we saw an effect of population of origin and TB-co-infection status, but no effect of the genetic variants we tested (table 5). We also analysed the effect of allelic variation on immune reconstitution following initiation of HAART, as measured by the response in CD4 count over the course of 48 weeks. Again, whilst observing an effect of time and baseline CD4 count on CD4 count during HAART, we found no effect of *FCGR2C*, *FCGR3A*, *FCGR3B* copy number and HNA1 allelic status on immune reconstitution (table 6).

**Table 5:** Tests of association of genotype with HIV load pre-HAART n=684.

**Genotype**	***FCGR3A* copy number**	***FCGR3A* copy number**	***FCGR3B* copy number**	***FCGR3B* copy number**	**HNA1 ratio**	**HNA1 ratio**	***FCGR2C* copy number**	***FCGR2C* copy number**
**Covariate**	**beta coefficient (95%CI) (copies/mL)**	**P value**	**beta coefficient (95%CI) (copies/mL)**	**P value**	**beta coefficient (95%CI) (copies/mL)**	**P value**	**beta coefficient (95%CI) (copies/mL)**	**P value**
Population (Ethiopian=1, Tanzanian=2)	-0.58 (-0.88,-0.28)	<0.001	-0.58 (-0.88,-0.28)	<0.001	-0.52 (-0.84,-0.20)	0.001	-0.57 (-0.87,-0.27)	<0.001
No TB Co-infection	-0.53 (-0.79,-0.27)	<0.001	-0.54 (-0.80,-0.27)	<0.001	-0.54 (-0.81,-0.28)	<0.001	-0.53 (-0.79,-0.27)	<0.001
CD4 count	-0.003 (-0.006, -0.001)	0.01	-0.003 (-0.006, -0.001)	0.007	-0.003 (-0.006, -0.001)	0.007	-0.003 (-0.006, -0.001)	0.008
Copy number	0.084 (-0.27, 0.43)	0.64	0.056 (-0.13, 0.24)	0.56	0.19 (-0.21, 0.58)	0.35	0.142 (-0.20, 0.36)	0.58

**Table 6:** Tests of association of genotype with CD4 count during HAART n=1823.

**Genotype**	***FCGR3A* copy number**	***FCGR3A* copy number**	***FCGR3B* copy number**	***FCGR3B* copy number**	**HNA1 ratio**	**HNA1 ratio**	***FCGR2C* copy number**	***FCGR2C* copy number**	
**Statistic**	**beta coefficient** (**95%CI**) (cells/mm^3^)	**P value**	**beta coefficient** (**95%CI**) (cells/mm^3^)	**P value**	**beta coefficient** (**95%CI**) (cells/mm^3^)	**P value**	**beta coefficient** (**95%CI**) (cells/mm^3^)		**P value**
Time after HAART initiation (weeks)	2.59 (2.36,2.82)	<0.001	2.59 (2.36,2.82)	<0.001	2.59 (2.36,2.82)	<0.001	2.60 (2.36,2.84)		<0.001
Baseline CD4 (cells/mm^3^)	0.86 (0.75, 0.97)	<0.001	0.86 (0.75,0.97)	<0.001	0.86 (0.75,0.97)	<0.001	0.86 (0.74,0.97)		<0.001
Population (Ethiopian=1, Tanzanian=2)	16.15 (2.17, 30.1)	0.024	15.80 (1.74,29.85)	0.028	17.97 (3.43,32.5)	0.02	16.16 (1.98,30.3)		0.03
No TB Co-infection	14.98 (2.05, 27.91	0.023	14.58 (1.59,27.56)	0.028	14.81 (1.89,27.7)	0.03	15.49 (2.37,28.9)		0.02
copy number	6.88 (-9.95,23.71)	0.423	-2.47 (-12.25,7.31)	0.621	-8.74 (-28.15,10.68)	0.38	-2.25 (-15.4,10.91)		0.74

It is known that the allelic sequence and copy number variation of Fcγ receptor genes determine the expression profile, activatory/inhibitory balance, and IgG affinity of the Fc receptor repertoire of each individual. Given the known importance of Fc genetic variation on antibody mediated immune responses we hypothesised that allelic and copy number variation might be associated with baseline viral load (HIV load prior to HAART administration) and progression (CD4 count during HAART treatment). For the two African populations studied, there was no evidence of association with the variants we have examined. The lack of association of Fc receptor genetic variation with HIV progression and baseline viral load would support studies in primate challenge models suggesting that non-neutralising antibodies provide limited or no protection against HIV [30]. However our data and primate challenge data contrasts with data from trials such as the RV144 trial which indicated a role for non-neutralising antibodies in mediating the protection found in 31% of their patients [8]. Alternatively, it may be that epistatic interactions between different FCGR alleles and IgG allotypes are important in host control of HIV. Indeed, epistasis has been described for KIR/HLA-C mediated control of HIV [31]. Larger epidemiological studies combined with functional approaches are needed to provide the power to test this possibility in a thorough manner.
